# The Utility of Cone Beam Computed Tomography Scans in Diagnosing and Treating Anterior Lesions of the Maxilla and Mandible

**DOI:** 10.7759/cureus.52804

**Published:** 2024-01-23

**Authors:** Kavya Shankar Muttanahally, Samantha Sheppard, Sumit Yadav, Aditya Tadinada

**Affiliations:** 1 Oral and Maxillofacial Radiology, Department of Growth and Development, University of Nebraska Medical Center, Lincoln, USA; 2 Department of General Dentistry, University of Connecticut, Farmington, USA; 3 Department of Growth and Development, University of Nebraska Medical Center, Lincoln, USA; 4 Department of Oral and Maxillofacial Radiology, University of Connecticut, Farmington, USA

**Keywords:** periapical lesions, anterior mandible, anterior maxilla, cone-beam computed tomography (cbct), digital panoramic radiographs

## Abstract

Background: The standard screening protocol for radiographic examination in dentistry as per the American Dental Association recommendations is a panoramic radiograph (PAN) and four horizontal bitewings. PAN inherently suffers from several shortcomings like the superimposition of anatomic structures, especially of the cervical spine that obscures a significant portion of the anterior maxilla and mandible. This region has a significant amount of pathology that is not adequately imaged. Three-dimensional (3D) imaging provides circumferential information on the area of interest and adds value to the diagnosis and treatment planning of pathology, especially in the anterior maxilla and mandible. However, there is not an adequate number of well-designed studies that articulate the true value addition of 3D imaging for the evaluation of this region.

Objectives: The objective of this study is to evaluate the value addition of 3D imaging in diagnosing pathologies in the anterior maxilla and mandible when compared to two-dimensional PAN.

Materials and methods: A total of 25 cases that had a diagnosis of anterior pathology and had both a PAN and a cone beam computed tomography (CBCT) scan were collected for this study. An institutional review board approval to retrospectively evaluate these data was obtained. The PAN and CBCT scans were randomly evaluated by a second-year dental student, an oral and maxillofacial radiology resident in training, and a board-certified oral radiologist. The scans were evaluated using a three-point modified Likert scale, where 1 represents "not visible or clear," 2 represents "visible but not clear," and 3 represents "visible and clear." The lesions were evaluated for characteristics like lesion location, size & shape, internal contents, borders of the lesion, cortical integrity, locularity, and effect on adjacent structures like root resorption. After the evaluation was completed, a comparison of the lesion diagnosis was done with histopathology to confirm the diagnosis. The evaluators were also asked to comment on the specific feature that 3D imaging provided that added value to the case. Kappa analysis was done to evaluate inter-operator reliability.

Results: PAN demonstrated significantly lower efficacy in identifying and diagnosing lesions. Only 56% of cases were analyzed using PAN, with 44% deemed undetectable or poorly visualized. These challenging cases necessitated CBCT scans for accurate diagnosis, which successfully diagnosed all 25 cases. The p-value of 0.0002 for PAN implies a highly significant difference from histopathology, suggesting the distinctions are not due to chance. Conversely, the p-value of 0.3273 for CBCT implies that observed differences may be random, lacking sufficient evidence to reject the null hypothesis. CBCT scans consistently outperformed PAN in visualizing various lesion characteristics, underscoring their superior diagnostic capabilities.

Conclusions: In this study, with a small sample size, 3D imaging provided a significant value addition to the diagnosis and treatment planning by providing additional information regarding the location, extent, internal content, and effect on adjacent structures. The practical implications for clinical settings, along with comparisons to current literature, underscore the study's distinctiveness.

## Introduction

In the field of oral and maxillofacial radiology, two-dimensional (2D) imaging, including extra-orals such as panoramic radiographs (PAN) and intraoral radiographs like bitewings, is commonly employed. PAN offers an overview of dental arches, providing detailed views of anatomical structures such as maxillary sinuses, temporomandibular joints (TMJ), and hyoid bone. However, PAN has limitations in evaluating specific anatomical structures and lacks the ability to display three-dimensional (3D) relationships [1-3].

In the United States, the standard screening protocol for radiographic examination in dentistry as per the American Dental Association (ADA) recommendations is a PAN and four horizontal bitewings adhering to established selection criteria [4]. Nevertheless, these methods have limitations, especially in detecting lesions and anatomical structures in the anterior jaws, primarily due to the ghosting of cervical vertebrae [5]. Image quality issues, such as superimposition of surrounding anatomy, double images, airway shadows, and patient positioning errors, can compromise PAN quality [2]. Additionally, PAN lacks the capability to evaluate 3D relationships [3].

Contrastingly, cone beam computed tomography (CBCT) imaging is gaining popularity for its accurate 3D imaging of hard tissue structures [6]. CBCT scans offer detailed views in axial, coronal, and sagittal planes, providing precise information about lesion size, shape, location, and extension, along with their relationship to surrounding tissues [3,7,8]. Compared to PAN, CBCT scans avoid superimposition and ghost images but may exhibit artifacts like motion and scattering due to metal, impacting image quality [3,9,10].

While PAN and intraoral radiographs are commonly taken, the advantages of CBCT in accurate 3D imaging are increasingly recognized. CBCT proves valuable in diagnosing oral and maxillofacial diseases. However, considerations such as cost and radiation dose are essential when choosing between CBCT and PAN [11]. The radiation dose in CBCT depends on various factors, and its effectiveness must be weighed against the significantly higher radiation dose associated with large-volume CBCT compared to PAN [12,13]. Apart from these, the clinicians must be aware of the anterior region. In comparison to PAN, the small to medium volume CBCT scan can provide the clinician with a good diagnosis and treatment plan and also it is a good modality for postoperative follow-up of large lesions comparison.

To date, there are only a few comparative studies between PAN and CBCT imaging that have focused on anterior lesions. Many anterior lesions are missed in PAN due to their image quality. Early diagnosis of any lesion plays a vital role in the treatment plan. Also, not every scan is read by a radiologist to arrive at an accurate diagnosis, so there is a high chance that clinicians may miss out on lesions in the anterior region in PAN [3]. Even if they can identify it, it is hard to know the extent of the lesions. Therefore, the objective of this study is to evaluate the value addition of 3D CBCT imaging in diagnosing pathology in the anterior maxilla and mandible when compared to 2D PAN.

## Materials and methods

Case selection

In this study, we examined 25 patients with anterior pathology. Each case included both PAN images and CBCT scans for a comprehensive examination. The total participants in the study were 25, with 17 females and eight males. Their ages ranged from 18 to 86 years, and the average age across all participants was 53.18 years. Approval from the University of Connecticut's Institutional Review Board (IRB) with reference number 22X-138-1 was secured for the retrospective data analysis. The imaging data were retrieved from the Picture Archiving and Communication System (PACS) within the Department of Oral and Maxillofacial Radiology at the University of Connecticut School of Dental Medicine. The selection criteria are mentioned in Table 1.

**Table 1:** Selection criteria CBCT: cone beam computed tomography.

Inclusion criteria	Exclusion criteria
1. Patients with lesions from canine to canine in the maxilla and/or mandible	Soft tissue lesions in the oral and maxillofacial region
2. Patients with both panoramic and CBCT scans	Lesions in the premolar and molar region
3. Patients with definitive histopathological diagnosis	Poor quality panoramic and CBCT scans

Radiographic interpretation

PAN images were saved as JPEG (Joint Photographic Experts Group) files and CBCT volumes as DICOM (Digital Imaging and Communications in Medicine) files, with all images and volumes undergoing deidentification. PAN images were examined using the MiPACS Dental Enterprise Viewer 3.1.1404 (Medicor Imaging, Charlotte, NC), and CBCT volumes were reviewed with Invivo 5.4.5 (Anatomage, San Jose, CA).

Observers conducted their assessments in a dimly lit room, utilizing a monitor with a resolution of at least 1650 × 1050 pixels. They were given the flexibility to navigate and manipulate images, adjusting magnification, brightness, and contrast. Additionally, on CBCT images, observers could generate volume renders and custom sections. Importantly, no clinical information was disclosed, and there were no time constraints during the review process.

The PAN images and CBCT scans were assessed independently by a board-certified oral radiologist, an oral and maxillofacial radiology resident in training, and a second-year dental student. Before the study, comprehensive training was provided to the second-year dental student for the assessment of CBCT and panoramic images. Utilizing a three-point modified Likert scale (Table 2), the scans were evaluated for lesion characteristics (Table 3).

**Table 2:** Three-point modified Likert scale

Three-point modified Likert scale
Not visible or clear
Visible but not clear
Visible and clear

**Table 3:** The anterior lesions were evaluated for characteristics

Lesions were analyzed based on the below features
1. Lesion location
2. Size
3. Shape
4. Internal contents
5. Borders of the lesion
6. Cortical integrity
7. Locularity
8. Effect on adjacent structures like root resorption

Post-evaluation, a comparison of the lesion diagnosis was made with histopathology to verify accuracy. Evaluators were also invited to share insights into specific features provided by 3D imaging that contributed value to the case. Interoperator reliability was determined using Kappa analysis.

## Results

The radiographic evaluation of the 25 cases was completed by three individuals, namely, a dental student, an oral and maxillofacial radiology resident in training (KM), and a board-certified oral and maxillofacial radiologist (AT). PAN demonstrated significantly lower efficacy in identifying and diagnosing lesions. Only 56% of cases were analyzed using PAN, with 44% deemed undetectable or poorly visualized. These challenging cases necessitated CBCT scans for accurate diagnosis, which successfully diagnosed all 25 cases. The p-value of 0.0002 for PAN implies a highly significant difference from histopathology, suggesting the distinctions are not due to chance. Conversely, the p-value of 0.3273 for CBCT implies that observed differences may be random, lacking sufficient evidence to reject the null hypothesis. CBCT scans consistently outperformed PAN in visualizing various lesion characteristics, underscoring their superior diagnostic capabilities.

First, each participant was asked to rank on a Likert scale of 1-3 if different radiographic characteristics of the lesion were visualized on a PAN compared with a CBCT scan. Table 4 shows the mean score the evaluators chose for visualizing each of the following characteristics with each modality. The results suggest that visualizing cortical integrity is significantly improved when evaluating with a CBCT rather than a PAN. Additionally, lesion borders, locularity, and internal contents are better visualized. These are all important characteristics in the diagnosis of intraosseous lesions.

**Table 4:** Radiographic characteristics evaluation results using a Likert scale 1. Scores are based on a Likert scale of 1 to 3, where 1 indicates a lower rating and 3 indicates a higher or more favorable rating. 2. The results consistently favor cone beam computed tomography (CBCT) over panoramic radiographs across all evaluated criteria, suggesting superior performance in assessing radiographic characteristics.

	Lesion location	Size	Shape	Internal contents	Borders of lesion	Cortical integrity	Locularity	Effect on adjacent structures
Panoramic radiograph	2.08	1.96	1.92	1.72	1.68	1.04	1.58	1.61
Cone beam computed tomography	3	3	3	3	3	3	3	3

Secondly, the evaluators were asked to list a differential diagnosis for the lesion provided, with a PAN in comparison to a CBCT scan. Diagnostic accuracy was determined based on whether the condition listed by the evaluator was consistent with the histopathologic confirmed diagnosis. In this study, evaluating a CBCT improves diagnostic accuracy with statistical significance.

Lastly, dependent t-tests were performed on the results of the evaluation. This determined whether there was a statistically significant difference in diagnosing anterior interosseous lesions using a PAN in comparison to a histopathological diagnosis (gold standard) as well as a CBCT scan in comparison to a histopathological diagnosis. The findings are outlined in Table 5. There was a statistically significant difference in diagnostic accuracy when comparing PAN and histopathologic diagnosis (gold standard). This suggests that histopathological diagnosis is significantly more accurate in diagnosis, which is an expected finding. However, there was not a statistically significant difference in diagnostic accuracy when comparing CBCT scans and histopathologic diagnosis (gold standard). This suggests that CBCT scans have high diagnostic accuracy.

**Table 5:** Dependent t-test results: PAN vs. CBCT vs. histopathological diagnosis PAN rejects the null hypothesis. CBCT fails to reject the null hypothesis. PAN: panoramic radiograph; CBCT: cone beam computed tomography.

	Panoramic radiograph	CBCT	Histopathology
Mean	0.56	0.96	1
Variance	0.2567	0.04	0
t stat	4.34	1	
P (T <= t)	0.0002	0.3273	

## Discussion

Clinicians are primarily concerned while evaluating jaw lesions, particularly with the location, size, shape, and boundary of the lesions, as well as their relationship with surrounding structures [13]. To assess any jaw lesions, choosing the appropriate radiological examination is essential to obtain comprehensive diagnostic information that can help in planning an appropriate treatment [3,13,14].

Our study evaluated the diagnostic accuracy of PAN and CBCT in the diagnosis of anterior maxillary and mandibular lesions. Three evaluators, including a dental student, an oral and maxillofacial radiology resident, and a board-certified oral and maxillofacial radiologist, evaluated 25 cases and ranked the clarity of visualizing different radiographic characteristics using a Likert scale. We also compared the differential diagnosis for each lesion and compared it with the diagnostic accuracy based on histopathological confirmation.

A study conducted by Mao et al. [3] has shown that there are significant differences in the radiographical features of jaw lesions on CBCT compared to PAN. The diagnostic accuracy and clinicians' confidence in evaluating CBCT has been demonstrated to be superior in comparison to PAN. These results can assist clinicians in making decisions about choosing appropriate imaging modalities during surgical planning or follow-up [15].

**Figure 1:**
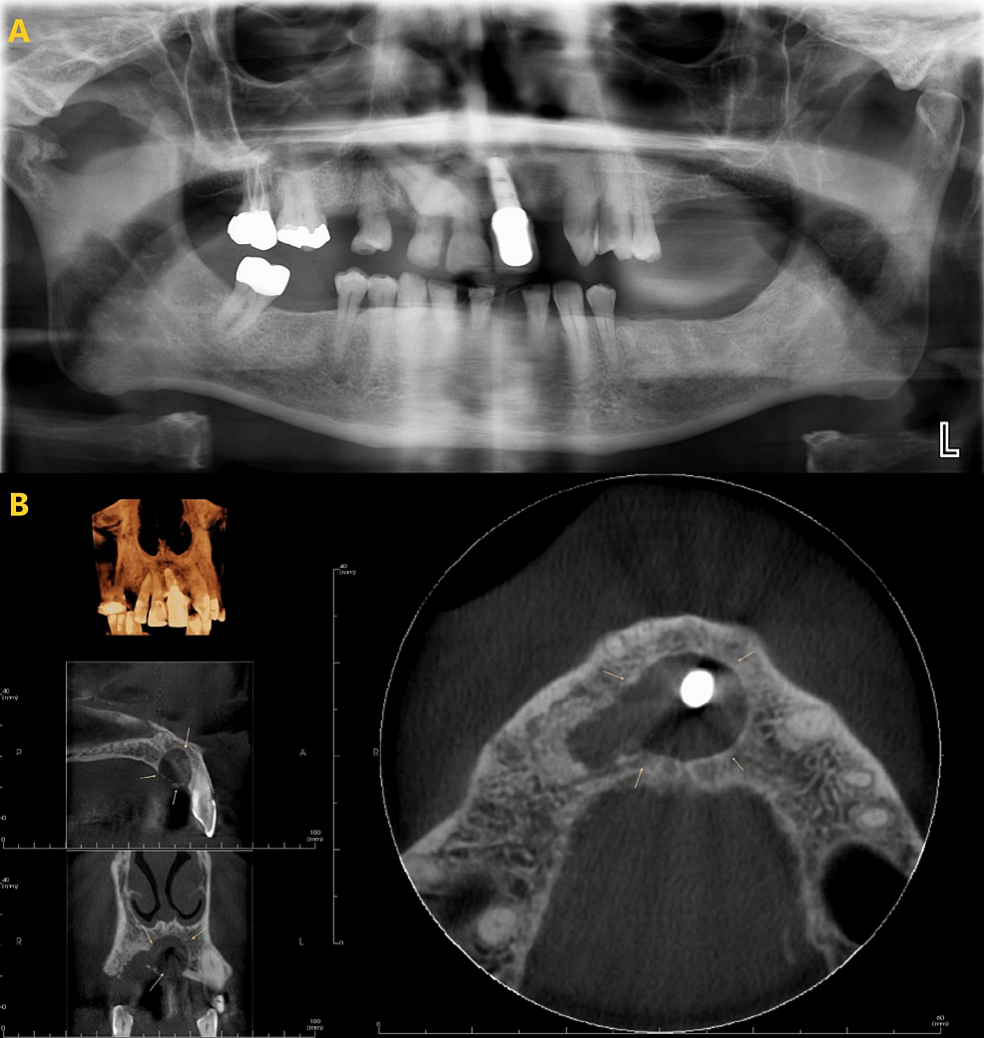
A periapical cyst, associated with the implant at site 9, was distinctly visualized in both PAN and CBCT. However, CBCT (B) offered enhanced clarity in assessing the size, shape, boundaries of expansion (marked in yellow arrows), and discontinuity of the cortex compared to PAN (A). PAN: panoramic radiograph; CBCT: cone beam computed tomography.

The results showed that CBCT (Figure 1B) scans significantly improved the visualization of cortical integrity, lesion borders, locularity, and internal contents of intraosseous lesions compared to PAN (Figure 1A).

**Figure 2:**
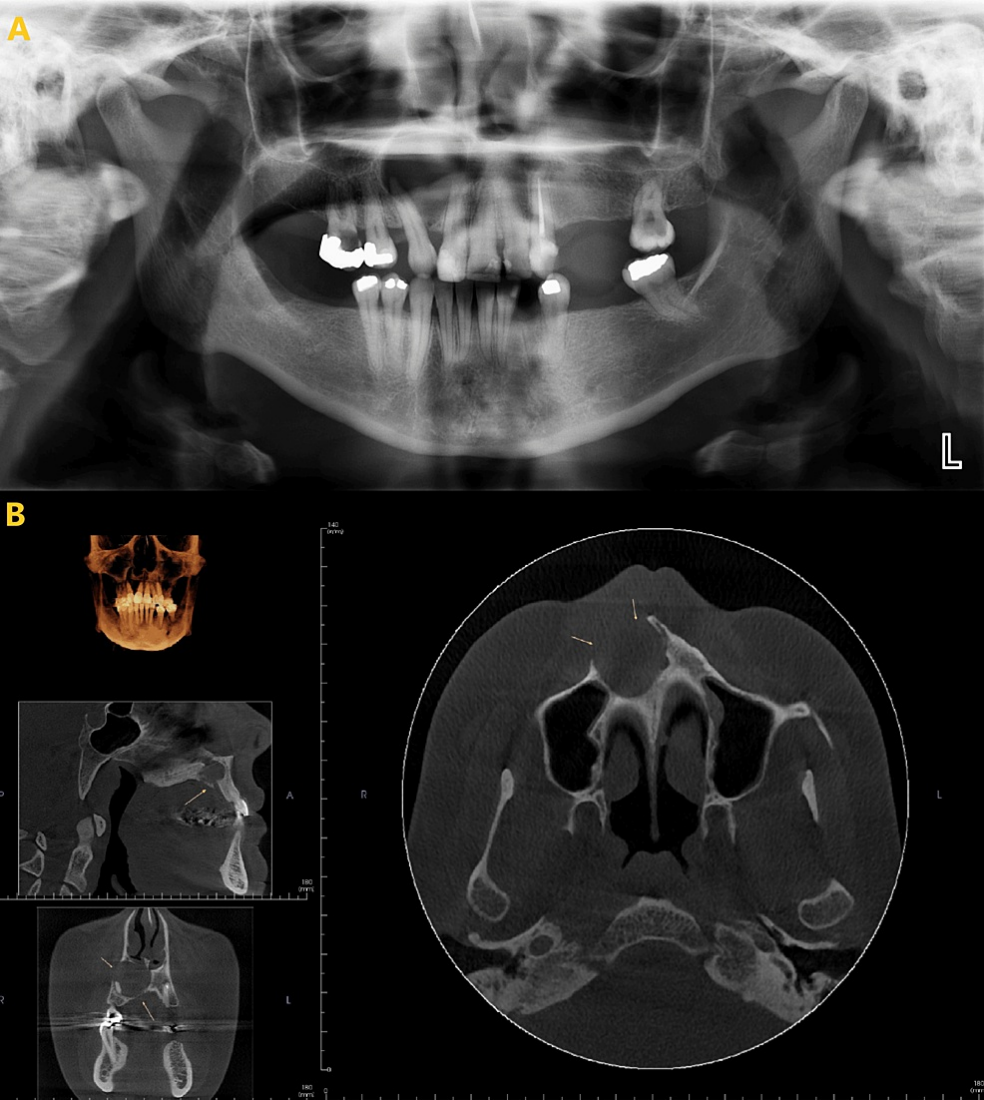
A large radiolucency in the anterior aspect was observed in both PAN and CBCT. However, CBCT (B) provided enhanced visualization, offering clearer details regarding the location, size, shape, and discontinuity of the facial cortex (marked in yellow arrows) compared to PAN (A). PAN: panoramic radiograph; CBCT: cone beam computed tomography.

**Figure 3:**
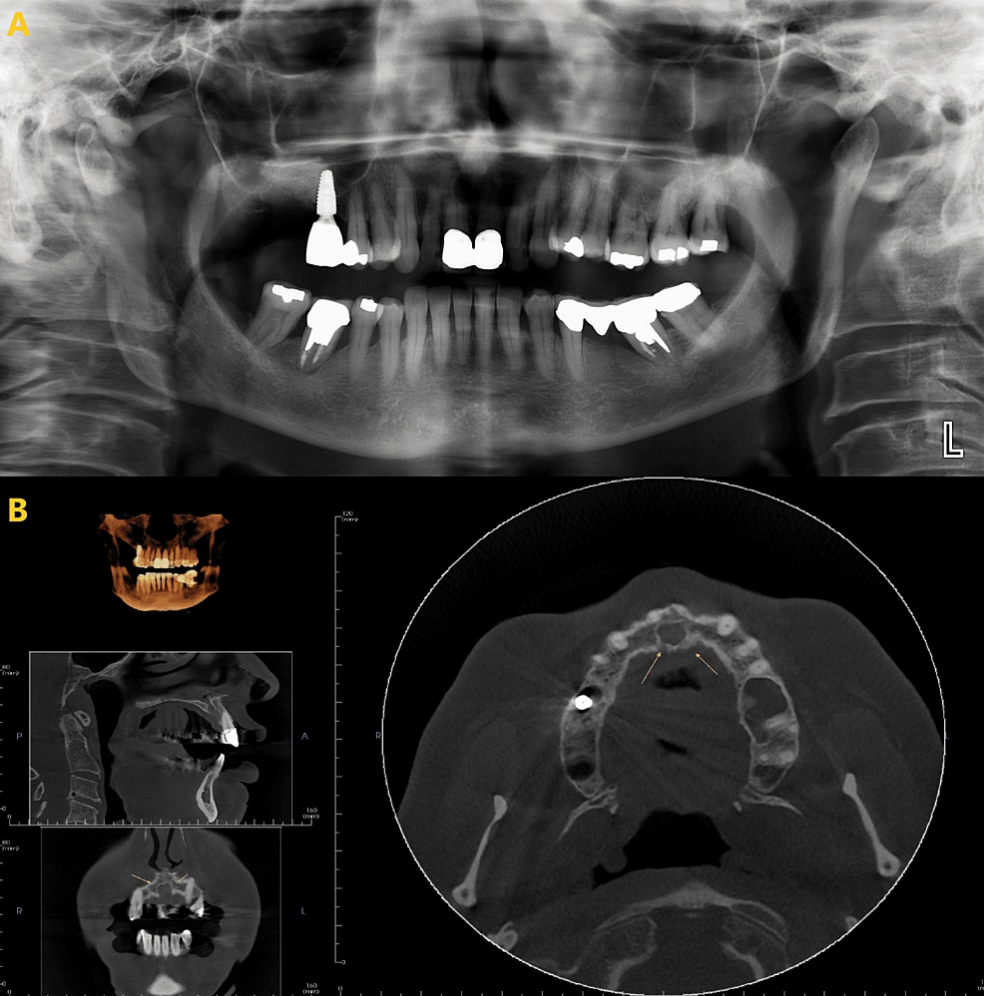
Nasopalatine canal cyst. PAN (A) lacked clarity in size, shape, and measurement, while CBCT (B) provided precise details (marked in yellow arrows). PAN: panoramic radiograph; CBCT: cone beam computed tomography.

This finding suggests that CBCT is a better imaging modality than PAN for detecting and diagnosing, especially the anterior region. CBCT (Figures 2B, 3B) provides more detailed images and 3D reconstruction of the bone, which allows for better visualization of the lesion characteristics. PAN, on the other hand, only provides a 2D image, along with it there is also the ghost image of the C-spine obscuring the anterior aspect making it more difficult to assess the border, shape, and size of the lesion accurately (Figures 2A, 3A). There were some differences in the comparison of imaging features between PAN and CBCT compared to the most recent report from Lim et al. [16]. The strongest agreement between the two modalities was observed in lesion shape, border definition, border cortication, internal contents, and tooth displacement. The differences observed in lesion shape between PAN and CBCT may be attributed to CBCT's ability to display the morphology of lesions from multiple slices and various views. This is particularly important as different morphological characteristics often guide differential diagnosis [17-19].

Dependent t-tests showed that there was a statistically significant difference in diagnostic accuracy between PAN and histopathological diagnosis. This finding suggests that histopathological diagnosis is more accurate than PAN in diagnosing intraosseous lesions. This is not unexpected since histopathology provides a definitive diagnosis based on the examination of the lesion tissue. However, there was no statistically significant difference in diagnostic accuracy between CBCT and histopathological diagnosis. This finding suggests that CBCT is a highly accurate imaging modality for diagnosing intraosseous lesions, which is similar to the previous study conducted by Mao et al. [3]. CBCT has been demonstrated as an ideal imaging modality for multiple dental applications, including implant site assessment, evaluating the paranasal sinuses, the neck region, and airway analyses, and has the unique advantage of being a low-dose and high-resolution imaging modality compared to both the 2D PAN and the 3D multislice CT scan.

In summary, the study strongly supports the superior diagnostic effectiveness of CBCT in comparison to PAN for imaging anterior jaw lesions. CBCT's 3D capabilities allow for a more detailed assessment of corticated borders, anatomic boundary expansion, cortical thinning, cortical destruction, and root resorption, especially in the anterior regions of both jaws and the maxilla. The identified higher diagnostic accuracy of CBCT, coupled with the elevated confidence reported by radiologists, underscores its pivotal role in advancing the precision of anterior jaw lesion assessments [20-22]. However, histopathology remains the gold standard for definitive diagnosis.

Limitations

This study makes valuable strides in unraveling the diagnostic advantages of 3D CBCT for anterior lesions. However, it is crucial to recognize and address certain inherent limitations. While the sample size is suitable for the study's specific objectives, caution is advised when applying these findings to larger and more diverse populations. The retrospective design, while offering insights, introduces potential biases that should be acknowledged. The diverse expertise levels among evaluators, ranging from a dental student to a certified radiologist, enrich the study's comprehensiveness but may introduce variability. Relying on histopathology for confirmation aligns with standard practices, yet the inherent uncertainties in this diagnostic process should be acknowledged. The study's focused examination of specific anterior lesions provides depth but may limit immediate generalizability to a broader spectrum of dental pathologies.

## Conclusions

Based on the results of this study, it can be concluded that CBCT is superior to PAN in imaging anterior lesions in the jaw. CBCT has the advantage of providing a 3D view of the lesion, which enables a more accurate assessment of the integrity of the corticated borders, expansion of surrounding anatomic boundaries, cortical thinning, cortical destruction, and root resorption, especially in the anterior regions of both jaws and in the maxilla. CBCT also has a higher diagnostic accuracy compared to PAN and radiologists were more confident when using CBCT. These findings could be used to develop guidelines for imaging pathology in the jaw.
